# Ecological, Molecular, Histopathological and Public Health Implications of Zoonotic Metacercariae *Clinostomum complanatum* (Rudolphi, 1814) in the Endemic Anatolian Fish, *Alburnus escherichii*

**DOI:** 10.1002/vms3.70270

**Published:** 2025-03-17

**Authors:** Nurten Aydogdu, Simonetta Mattiucci, Marialetizia Palomba, Ali Aydogdu, Çiğdem Ürkü, H. Emre Yılmaz, I. Tülay Çağatay, Sadi Aksu

**Affiliations:** ^1^ Department of Hydrobiology, Graduate School of Natural and Applied Sciences Balıkesir University Balıkesır Turkey; ^2^ Department of Public Health and Infectious Diseases Section of Parasitology, Sapienza University of Rome Rome Italy; ^3^ Department of Ecological and Biological Sciences (DEB) Tuscia University Viterbo Italy; ^4^ Department of Aquatic Animal Diseases, Faculty of Veterinary Medicine Bursa Uludag University Bursa Turkey; ^5^ Faculty of Aquatic Sciences, Department of Fish Diseases Istanbul University Istanbul Turkey; ^6^ Department of Basic Sciences, Faculty of Fisheries Akdeniz University Antalya Turkey; ^7^ Vocational School of Health Services Eskişehir Osmangazi University Eskişehir Turkey

**Keywords:** anatolian fish, *Clinostomum complanatum*, metacercariae, zoonotic fluke

## Abstract

**Aims:**

Clinostomid metacercariae infect a wide range of freshwater fish species, posing a zoonotic risk to human health when consumed raw or undercooked, potentially leading to Halzoun disease. Although these parasites are generally considered a health threat in Asian countries, they are also present in various regions of Türkiye and have been found in 12 different freshwater fish species commonly consumed in local cultures. However, their presence has not been reported in the endemic Anatolian fish, *Alburnus escherichii*. This study aims to determine the presence of clinostomid species in *A. escherichii* from the Sarısu stream in Eskişehir, Türkiye.

**Methods and Results:**

In addition to using a combination of ecological, morphological, molecular and histopathological approaches, our findings revealed the presence of *Clinostomum complanatum* within the branchial and buccal cavities of *A. escherichii*. Further histopathological examination revealed encysted metacercariae of *C. complanatum* invading the host tissues encapsulated within a thin fibrotic layer and accompanied by moderate inflammatory cell infiltration and degenerative changes in muscle cells.

**Conclusions:**

These results provide new insights into the geographical and host distribution as well as histopathological impacts of *C. complanatum* metacercariae in teleost species.

## Graphical abstract

• *Clinostomum complanatum* metacercariae, which has been reported in 23 fish species worldwide and 12 in Türkiye to date, continues to infect local and endemic fish species and will persist in the future.

• Considering that *Alburnus escherichii* and other fish species in which this parasite has been recorded in Türkiye are used in traditional local cuisine, these fish species currently pose a possible zoonotic risk to humans.

• Important zoonotic helminths found in fish in Türkiye are not sufficiently known, and very few studies have been conducted to date on monitoring zoonotic parasites, and more studies are needed.

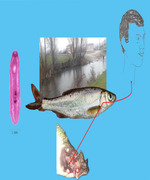

## 1 Introduction

According to our current knowledge, various freshwater fish and amphibian species serve as second intermediate hosts for the species belonging to the genus *Clinostomum* Leidy, 1856, which is represented by 58 species, and a wide variety of piscivorous birds serve as definitive hosts (Jithila and Prasadan 2019). Species of this genus have a very wide geographical distribution (Jithila and Prasadan 2019). *Clinostomum complanatum* (Rudolphi, 1814) Braun, 1899, widely distributed in the Western Palearctic region, is a well‐known representative of the genus *Clinostomum* (Matthews and Cribb 1998). This parasite species has been recorded in freshwater fish distributed in many countries of the world (Çağatay et al. 2022). *C. complanatum* was recorded in Türkiye waters for the first time in 1988 (Özer 2021). After this date, it has also been reported from 12 fish species living in different habitats from Turkey (e.g., *Rutilus rutilus*, *Perca fluviatilis* and *Scardinius erythrophthalmus*) (Şimsek et al. 2018; Özer 2021; Çağatay et al. 2022).

After the first human infection with *C. complanatum* was reported in 1938 (Yamashita 1938), there are studies reporting that this parasite infects humans as its definitive host (see, e.g., Chung et al. 1995b). On the other hand, although *C. complanatum* has been reported in freshwater fish species (*Cyprinus carpio*, *Squalius cephalus* and *P. fluviatilis*) that are distributed across different regions in our country and many of which have economic significance value and serve as a food source for the local population, to the best of our knowledge, no human case of *Clinostomum* infection has been reported in Turkey. We think that the fish species named above have a possible zoonotic risk for consumers, as they are fish species used in traditional local cuisine. We also think that the studies investigating *Clinostomum* species that infect fish species commonly found in our country's freshwaters are insufficient. One of these fish species is *Alburnus escherichii* Steindachner, 1897. The genus *Alburnus* comprises 24 species, including 15 endemic to Türkiye. *A. escherichii*, originally described from Tabakhane stream (Ankara, Sakarya basin), is naturally distributed in Akarçay, Sakarya and Batı Karadeniz basins (Çiçek et al. 2018, 2020) and was translocated to Konya Endorheic basin. Recently, it has also been claimed to be distributed in the Antalya basin (Küçük et al. 2020). However, to date, although many studies have been conducted on the ichthyoparasitic fauna of *A. escherichii*, *C. complanatum* infection has not been recorded in the host fish (Ozbek and Oztürk 2010; Öztürk 2011, 2017, 2022; İnnal et al. 2010, 2007, 2016, 2020; Nejat et al. 2023). Therefore, the parasite fauna of this host fish is probably not fully characterized. First, the present study aimed to provide additional information on the clinostomid fauna of this host fish in Turkish waters: to determine how the prevalence, abundance and intensity of infection vary according to seasons, as well as with the size and sex of the host fish.

To date, of the 183 species of helminth parasites recorded in freshwater fish in Türkiye (Özer 2021), the histopathological changes caused by only 5 species in the tissues of fish individuals have been examined (Aydogan et al. 2018; Innal et al. 2019; Gul et al. 2020; İnnal et al. 2020a; Ozmen et al. 2021). Apart from these studies, there is no study conducted to determine the histopathological changes caused by almost all other helminth parasite species recorded in host fish specimens in Türkiye. Due to a lack of histopathological information regarding fish infected with yellow grub trematodes, the second aim of this study was to perform histopathological investigations of some tissues infected with *C. complanatum*.

Furthermore, due to the high morphological similarity among different clinostomid species infecting freshwater fish in recent years, advanced molecular techniques have been used to effectively distinguish these species (Caffara et al. 2020; Acosta et al. 2016; Dzikowski et al. 2004). In Türkiye, two studies were conducted on the morphological and molecular characterization of *C. complanatum*, which was recorded in only two freshwater fish species (Şimsek et al. 2018; Çağatay et al. 2022). However, clinostomid infections in different fish species are rarely investigated. Third, in this study, it was aimed to confirm the species identification of *C. complanatum*, isolated for the first time from *A. escherichii* and identified on the basis of morphological and molecular characteristics using the ITS region.

## 2 Materials and Methods

### 2.1 Study Area and Collection of Fish

**FIGURE 1:**
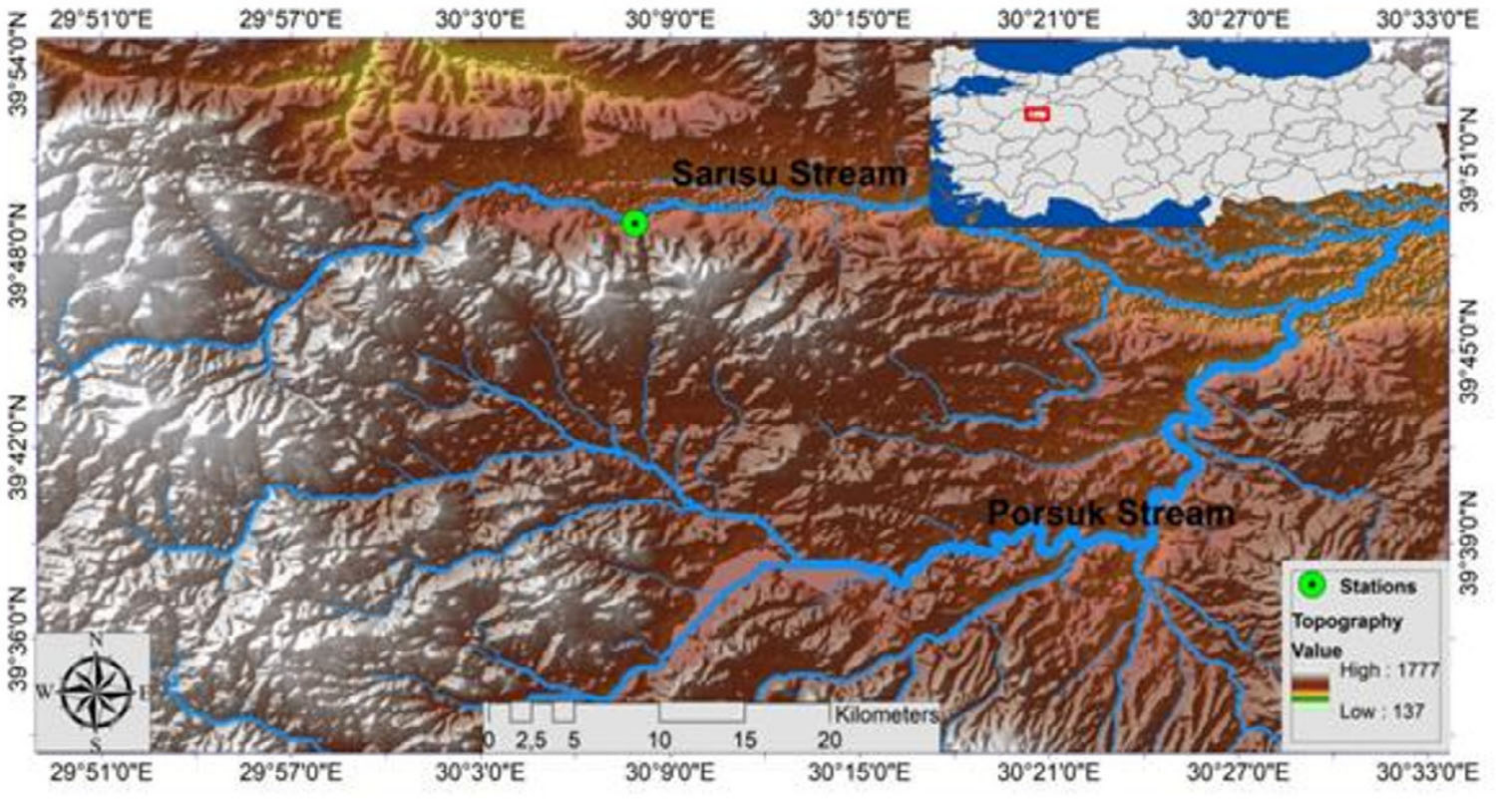
Sampling locality (green colour) of 216 specimens of *Alburnus escherichii* fished from Sarısu stream in Eskişehir between winter 2023 (February) and autumn 2023 (September).

Sarısu stream is an important source with a water temperature of at least 16°C, and it mixes with Porsuk stream from Eskişehir city centre. Sarısu River has two water sources that feed the Sarısu stream, the lowest water temperature of which is 16°C (Emiroğlu et al. 2016). It has been observed in previous studies that these resources show different feeding and habitat selection behaviours of both alien and local/endemic species interacting with these species (Emiroğlu et al. 2016). In the habitats where the sampling stations are located, endemic species other than the alien species that are the subject of the article, namely, *A. escherichii* and *Capoeta baliki*, have been reported in previous studies (Emiroğlu et al. 2016). A total of 216 *A. escherichii* were collected using electrofishing equipment (Samus 1000) from Sarısu stream in Eskişehir (Figure 1), between winter 2023 (February) and autumn 2023 (September), with seasonal intervals (once every 3 months). During each sampling campaign, 41–70 *A. escherichii* specimens were collected. The number of fish sampled per season is shown in Table 1. The specimens were transported alive to the laboratory in ventilated plastic tanks from the sampling sites in Sarısu stream. They were kept in 20‐L aerated aquaria and examined within 2–3 h. Fish species were euthanized by vertebral separation, measured for total length (cm) and divided into two groups based on length: the first group ranging from 6.00 to 9.99 ± 0.91 cm and the second group from 10.00 to 14.00 ± 1 cm. The sex of each fish was determined upon dissection; a total of 116 were females and 100 were males (Table 1).

**TABLE 1:** Distribution of infection value of *Clinostomum complanatum* metacercariae in *Alburnus escherichii* from Sarısu stream, Eskişehir, according to seasons, the length and the sex of the host fish.

		Examined fish number	Infected fish number	Prevalence (%)	Intensity mean ± SD	Abundance mean ± SD	Intensity range (min–max)
**Seasons**	Winter 2023	49	22	44.8	6.3 ± 6.8	2.8 ± 5.53	1–30
	Spring 2023	70	31	44.2	10.3 ± 9.5	4.5 ± 8.16	1–33
	Summer 2023	56	12	21.4	4.6 ± 8.1	1 ± 4.11	1–30
	Autumn 2023	41	8	19.5	4.1 ± 4.3	0.8 ± 2.42	1–13
**Fish sex groups**	Female	116	47	40.5	8.4 ± 8.8	3.4 ± 6.98	1–30
	Male	100	26	26	5.8 ± 7.3	1.5 ± 4.51	1–29
**Fish length (cm) ± SD**	6–9.90 ± 0.91	127	21	16.5	4.6 ± 7.1	0.7 ± 3.32	1–29
	10–14 ± 1	89	52	58.4	8.6 ± 8.6	5.0 ± 7.87	1–33

### 2.2 Parasitological Investigation

All collected fish were screened for the presence of *Clinostomum* metacercariae in various body parts, including the skin, fins, abdominal cavity, gills, eyes, buccal cavities and skeletal muscle using stereomicroscopes (SMZ 745 Nikon and SZ61 Olympus). When present metacercariae were carefully removed using dissection needles under stereomicroscopes. The exact location of the metacercariae was recorded for each infected fish. Data on the host fish were categorized according to the season of collection, host length and sex. Encysted metacercariae were transferred to saline solution and excysted by breaking the cyst wall with a fine needle. Isolated metacercariae were relaxed overnight in tap water before fixation in 5% formalin. Some living worms were stored in ethanol for morphological and molecular analyses. For morphological identification, some fixed metacercariae were stained with iron‐acetocarmine, differentiated with a mixture of hydrochloric acid and ethanol and washed in water. The worms were then dehydrated in a graded ethanol series (50%, 70% and 90% to absolute ethanol) before being placed in carbol‐xylene. Finally, samples were transferred to pure xylene, mounted in Canada balsam on a microslide and identified under an optical microscope according to diagnostic morphological keys of Matthews and Cribb (1998), Gibson et al. (2002), Caffara et al. (2014, 2020) and available references. Photomicrographs were captured using a photographic camera mounted on a Leica DMR microscope with phase contrast and an Olympus BX‐50 research microscope. For molecular identification, clinostomids were delivered to the Laboratory of Parasitology, Department of Public Health and Infection Diseases of ‘MS LAB (Eskisehir, Türkiye)’.

### 2.3 Molecular Identification

DNA from each individual was extracted using the EURx Tissue & Bacterial DNA purification kit (EURx, Poland) according to the manufacturer's instructions. PCR reactions were performed using the universal primers ITS5(5′‐GGAAGTAAAAGTCGTAACAAGG‐3′) and ITS4 (5′‐TCCTCCGCTTATTGATATATGC‐3′) (White et al. 1990). PCR reactions were performed in a total volume of 50 µL, containing 1XPCR Buffer, 2.5 µL MgCl_2_ (25 mM), 1 µL each primer (10 µM), 0.2 mM of dNTP mix, 0.5 U/µL Taq DNA polymerase and 4 ng of genomic DNA. PCR conditions were as follows: 5 min at 95°C, 35 cycles of 30 s at 94°C, 45 s at 60°C and 1 min at 72°C, 1 min at 72°C followed by 10 min at 72°C final extension step. The successful PCR products were purified, using Gene JET Gel Extraction and DNA Cleanup Micro Kit (Thermo, USA) according to the manufacturer's protocol and were Sanger sequenced in both directions by Medsantek Company (Istanbul, Türkiye). The obtained sequences were analysed, edited and assembled using CodonCode Aligner v6.0.2 (CodonCode Corporation) and ClustalW and compared with those available in GenBank using BLASTn.

Phylogenetic tree of the ITS region was constructed using Bayesian inference (BI) with MrBayes, v. 3.1.2 (Huelsenbeck et al. 2001). The Bayesian posterior probability analysis was performed using the MCMC algorithm, with four chains, 0.2 as the temperature of heated chains, 5,000,000 generations, with a subsampling frequency of 100 and a burn‐in fraction of 0.25. The saturation (ESS values) was monitored using Tracer v.1.7 (Rambaut 2018). Posterior probabilities were estimated and used to assess support for each branch. Values with a 0.90 posterior probability were considered well‐supported. *Diplostomum spathaceum* (JX986847.1) and *Euclinostomum heterostomum* (MT785768.1) were used as outgroups. Genetic distances were computed using the Kimura 2‐parameter (K2P) model with 1000 bootstrap re‐samplings, using MEGA Software, version 7.0. (Kumar et al. 2018).

### 2.4 Histopathological Studies

For histopathological studies, tissue samples taken from the buccal cavity affected by *C. complanatum* cysts were washed thoroughly with water and immediately fixed in 10% buffered formalin. After fixation, the tissue samples were gradually dehydrated with a graded series of ethanol series, cleaned in xylene and embedded in paraffin wax, according to the standard histological procedures. Thick sections of 4–5 µm were taken from all tissue samples with a microtome (Leica RM2125, Germany); the preparations were stained with Mayer's haematoxylin and eosin (H&E). Slides were examined under an Olympus BX‐51 light microscope equipped with an Olympus DP72 digital camera.

### 2.5 Statistical Analysis

Data on clinostomid species were categorized according to the seasons and the length and sex of the host fish. The levels of prevalence, mean intensity and abundance of infection, as defined by Bush et al. (1997), were calculated. Standard statistical computations (standard deviation) were carried out using Microsoft Excel (Microsoft Corporation 2000). Kruskal–Wallis tests were applied to find significant differences in the mean intensity of the parasite species for the host fish size and seasons. The Mann–Whitney *U*‐test was used to determine the association between the intensity of *C. complanatum* parasite and the host sex. A significance level of *p* ≤ 0.05 was used. All statistics analyses were performed using SPSS v. 28.

## 3 Results

### 3.1 General Infection Values of *C. complanatum* Metacercariae

**FIGURE 2:**
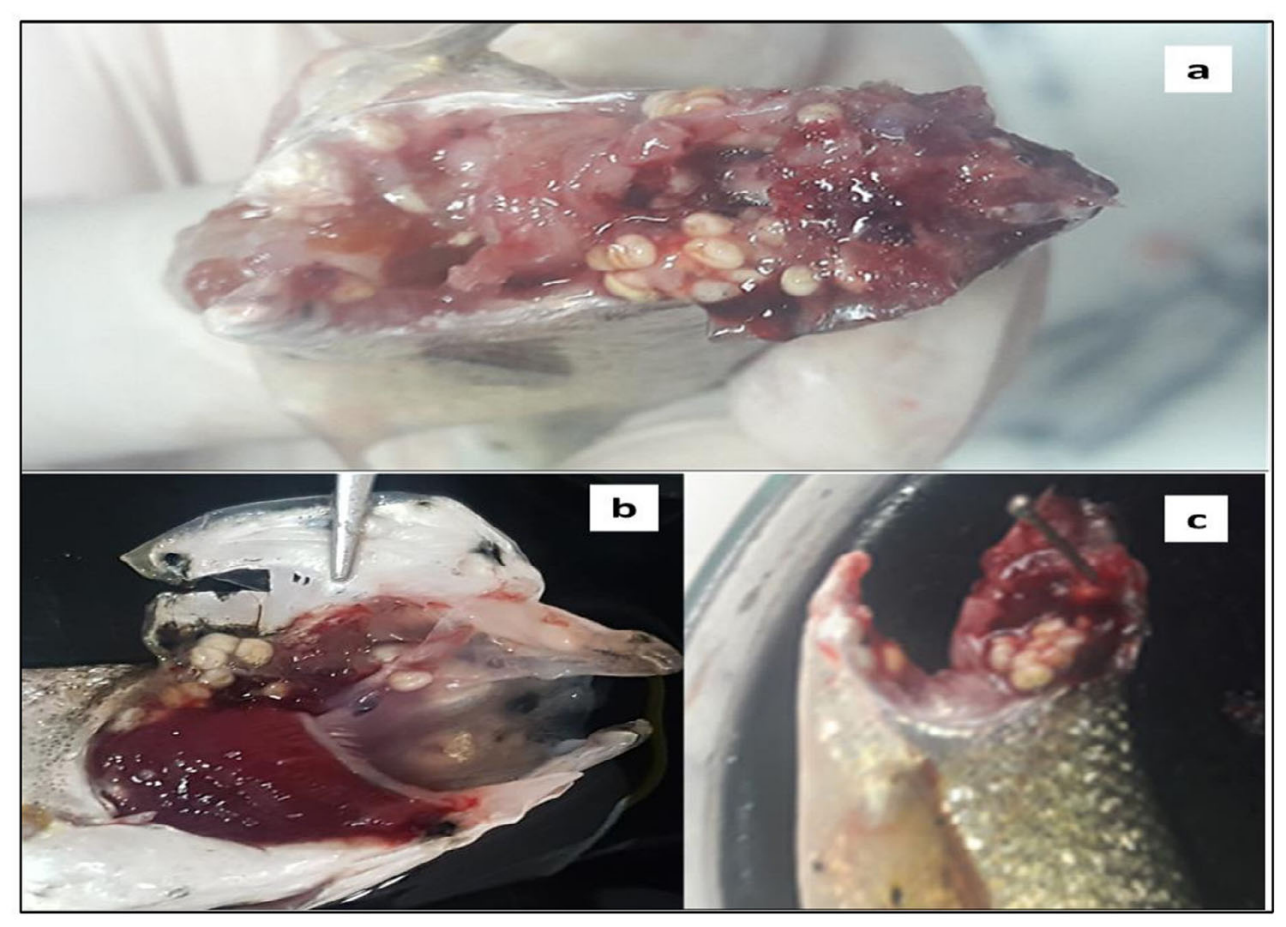
*Alburnus escherichii* infected with clinostomid encysted metacercariae: (a) the branchial cavity; (b and c) the buccal cavity.

**FIGURE 3:**
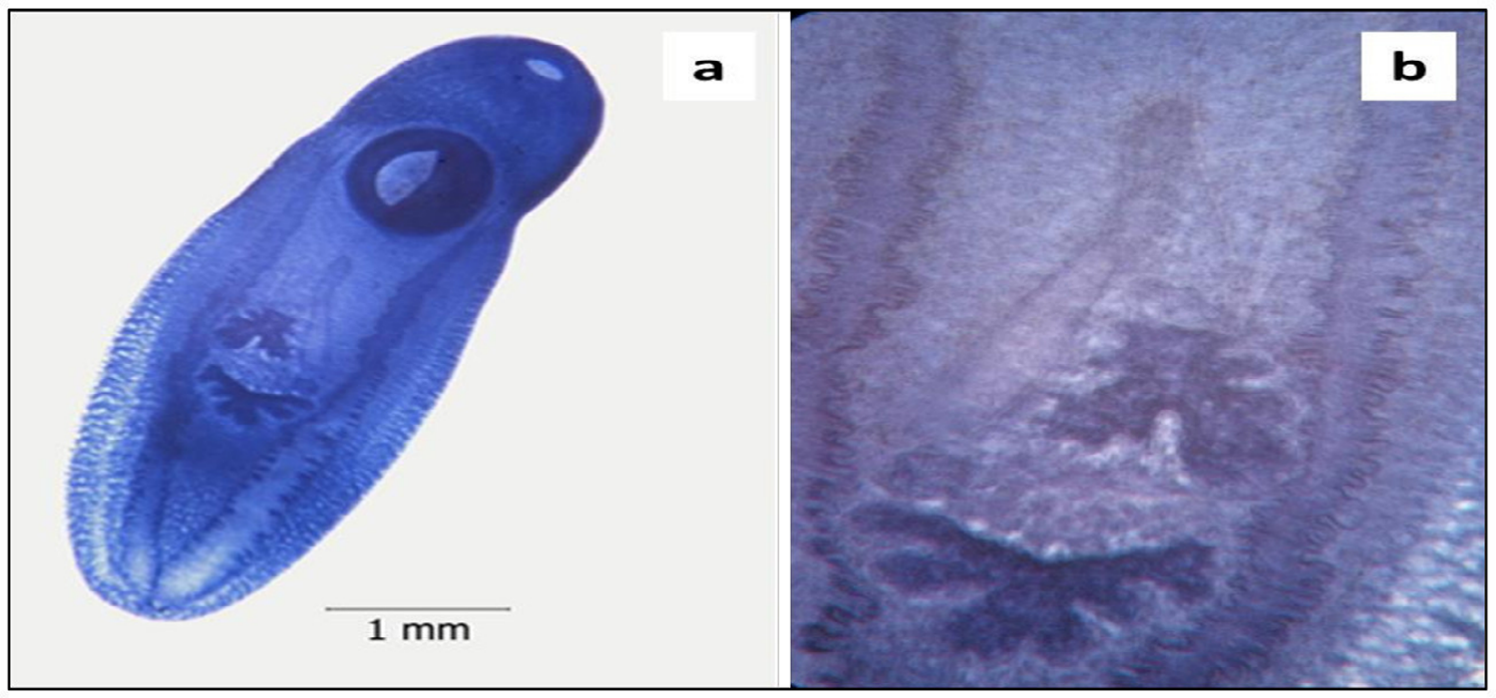
*Clinostomum complanatum* metacercariae from *Alburnus escherichii*: (a) Total view; (b) Genital complex x 80.

Overall, a total of 549 specimens of *C. complanatum* were found infecting the buccal cavity of 73 of the 216 examined fish (Figure 2a–c), resulting in an overall prevalence of 33.7%, (combining both sexes). The mean intensity was 7.5 ± 8.3, whereas the mean abundance was 2.5 ± 6.02. On the basis of detailed light microscopy studies on the morphology, the species was identified as *C. complanatum* (Figure 3a, b), then confirmed by molecular analysis.

### 3.2 Distribution and Infection Values of *C. complanatum* Metacercariae With Respect to Season

A definite seasonal effect was noted for *C. complanatum* with significant differences observed in infection parameters across seasons, as presented in Table 1. In detail, seasonal prevalence levels were recorded as follows: In winter, prevalence was 44.8%, gradually decreasing to 44.2% in spring, and further dropping to 21.4% in summer; the lowest prevalence level of 19.5% was detected in autumn. Both mean intensity and mean abundance peaked in spring (Table 1), and they reached their lowest values in autumn. Statistical analysis using the Mann–Whitney *U*‐test revealed significant differences between the number of parasites collected in summer compared to winter (*p* = 0.005, *U* = 1008.00), winter compared to autumn (*p* = 0.008, *U* = 728.00), spring compared to summer (*p* = 0.003, *U* = 1440) and spring compared to autumn (*p* = 0.004, *U* = 1030.00).

### 3.3 Distribution and Infection Values With *C. complanatum* Metacercariae by Fish Size

The specimens of *A. escherichii* within the size range of 6.00–14.00 ± 1.62 cm were analysed to investigate the relationship between the sizes of the fish host and the infection values with *C. complanatum* metacercariae (Table 1). A clear increase in the infection values was observed as the length of the host fish increased. Higher prevalence was noted in larger fish (58.4%) compared to those in smaller size classes (16.5%) (Table 1). Similarly, the mean intensity and mean abundance reached their maximum levels (8.6 ± 8.6, 5 ± 7.87, respectively) in larger size classes (Table 1). One *A. escherichii* with the size of 13 cm was the most infected by this metacercariae (33 individuals). However, a moderate (*r* = 0.534) and significant (*p* = 0.001) relationship was found between the number of *C. complanatum* metacercariae infections and the length of *A. escherichii*.

### 3.4 Distribution and Infection Values of *C. complanatum* Metacercariae With Respect to the Sex of Fish Host

Of the 216 specimens of *A. escherichii* collected, 116 were female and 100 were male (Table 1). Among the females, 47 were found to be infected by one or more clinostomid parasite species, resulting in a prevalence level of 40.5%. The mean intensity and mean abundance values in female individuals of *A. escherichii* were recorded as 8.4 ± 8.8 and 3.4 ± 6.98, respectively (Table 1). *C. complanatum* metacercariae were found in 26 out of 100 male fish examined, with prevalence, mean intensity and mean abundance of infection of 26%, 5.8 ± 7.3 and 1.5 ± 4.51, respectively. The prevalence, mean intensity and mean abundance values of infection were higher in females compared to males (Table 1). Moreover, there was a statistically significant sex‐related difference in the number of this species based on the Mann–Whitney *U*‐test (*p* = 0.011, *U* = 4815.5).

### 3.5 Molecular Identification of *C. complanatum* Metacercariae

**FIGURE 4:**
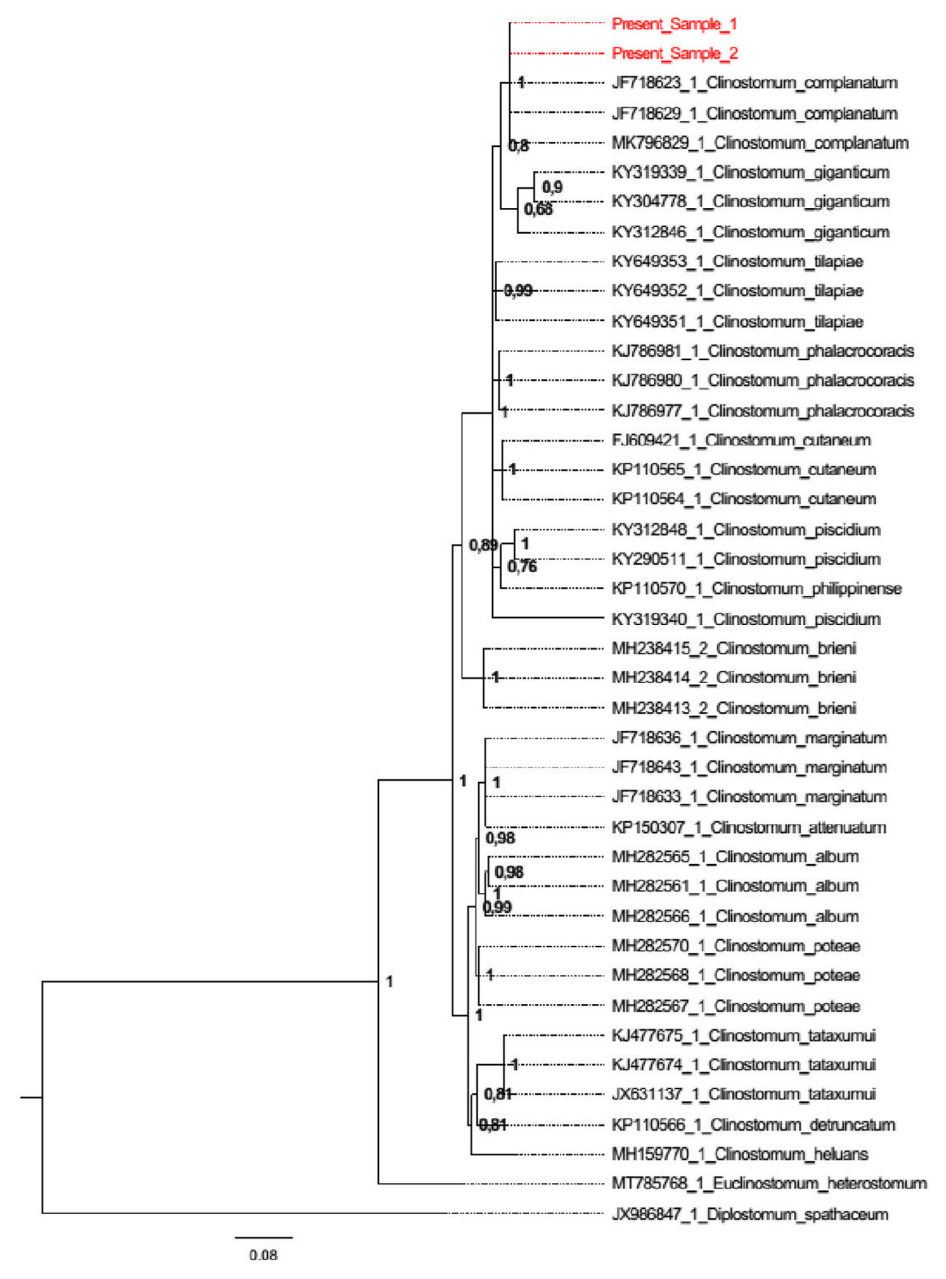
Cross‐section of the encysted *Clinostomum complanatum* metacercaria surrounded by the fibrotic connective tissue (a–c). Notice mild inflammatory cell infiltrations and two metacercariae are firmly attached to the host tissue by their ventral suckers (*), the excysted metacercaria is encircled by eosinophilic granular cells (d). (H&E).

The ITS gene region (1021 bp) of the parasites was successfully amplified. BLAST analyses showed a 100% similarity with *C. complanatum* (accession number, MK811210). The genetic distance showed that the closest species was *C. complanatum* (0.00%, JF718623, JF718629 and MK796829), whereas the most distant species was *C. piscidium* (0.11%, KY319340). As illustrated in Figure 4, the BI tree topology, inferred from the phylogenetic analysis of the sequences obtained from the ITS region of *C. complanatum* metacercariae, showed that they clustered within the same clade, supported by a high probability value (PP = 1.00), which also included sequences already deposited in the NCBI database as *C. complanatum*.

### 3.6 Histopathological Changes Caused by *C. complanatum* Metacercariae

**FIGURE 5:**
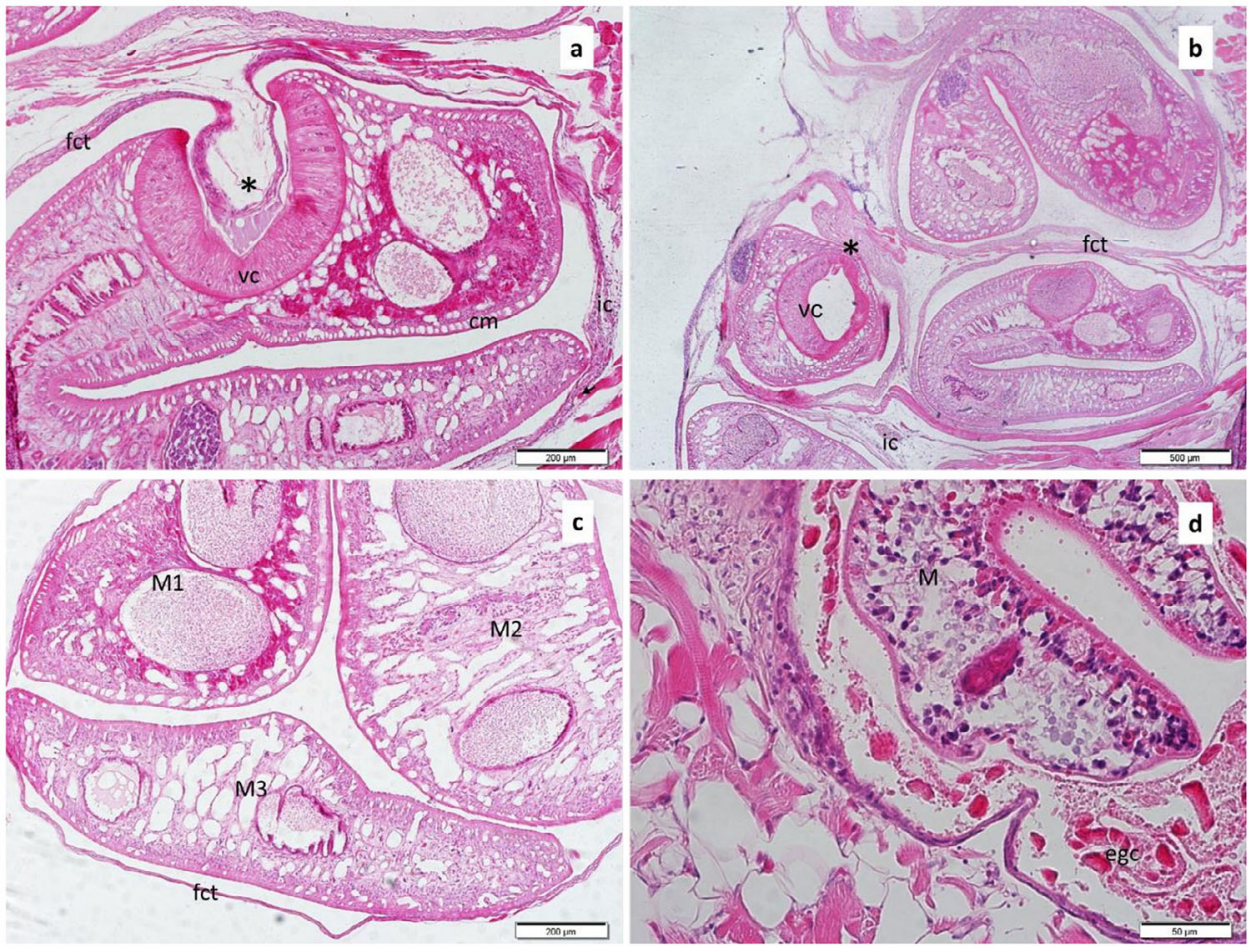
Cross‐section of tissues around excysted metacercariae (M). Degenerative changes (arrowed) in the muscle tissue around the excysted metacercariae (a and b). Notice inflammatory cells (IC) (c) and cluster cells (arrowed) (d). (H&E).

**FIGURE 6:**
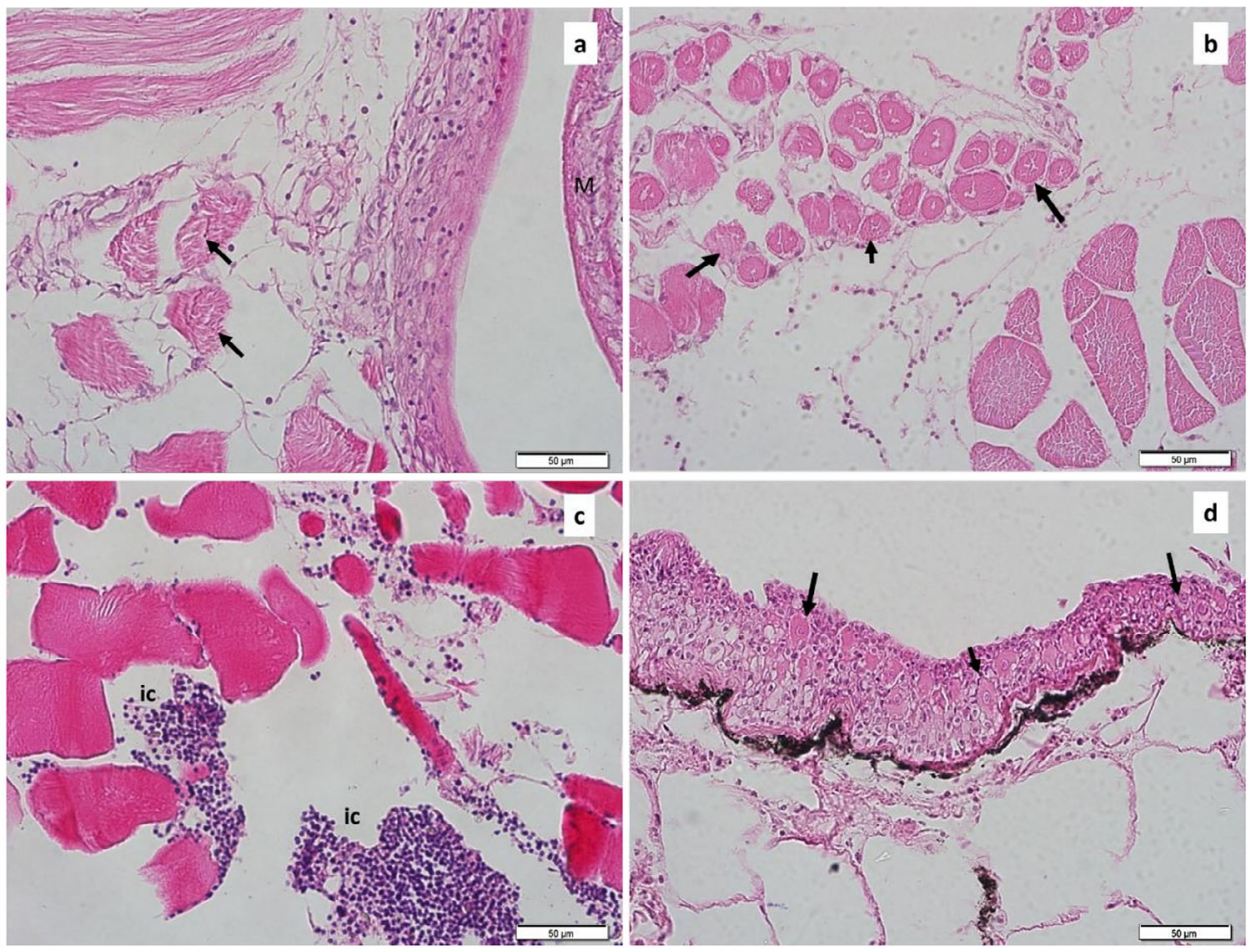
Phylogenetic tree from Bayesian inference on ITS sequences of *Clinostomum complanatum* obtained in the present study, with respect to the sequences of clinostomid species at the same gene locus available in GenBank.

Macroscopically, cystic metacercariae were seen inside round yellowish capsules; however, microscopically, the histological structure of *Clinostomum* metacercariae embedded in the muscles of the branchial cavity was revealed. Histologically, the cuticle and the hypodermis of the metacercariae, as well as the structure of the suckers that provide attachment to the host, were observed in the histological sections (Figure 5a, b). Two forms of metacercariae have been identified (encysted and excysted). Fibrotic connective tissue was noted around encysted metacercariae and mild inflammatory infiltrations were detected around this structure (Figure 5b). More than one metacercariae were observed in some encysted structures (Figure 5c). Additionally, in excysted forms, eosinophilic granular cell infiltrations (Figure 5d), degenerative changes in muscle tissue and the presence of cluster cells were noted (Figure 6a–d).

## 4 Discussion

To date, despite the nine ichthyohelmintological studies on fish fauna being conducted on the helminth parasites of *A. escherichii* (Özer 2021), *C. complanatum* metacercariae was not previously recorded. In this study, we reported for the first time, the infection with *C. complanatum* metacercariae in *A. escherichii* from Türkiye. *A. escherichii* represents a new host, thereby expanding the number of recorded host fish for this parasite to 13 specimens in Türkiye. In addition, we provided the first evaluation of the infection levels of clinostomid parasite species according to seasonality, sex and length classes of the fish host. Previous studies have reported that many freshwater fish species and ornamental fish, including various edible freshwater fish, act as potential intermediate hosts of *C. complanatum* (Jithila and Prasadan 2019). According to the existing literature, more than 25 fish species living in different localities around the world have been reported to serve as second intermediate hosts for *C. complanatum* (Jithila and Prasadan 2019; Çağatay et al. 2022). It has also been reported in 12 fish species living in different habitats across Türkiye (see, e.g., Özer 2021; Çağatay et al. 2022). Infection values of this parasite species have been previously reported in only eight fish species from Türkiye, namely, *P. fluviatilis*, *Sander lucioperca*, *Lepomis gibbosus*, *R. rutilus*, *S. erythrophthalmus*, *S. cephalus*, *Rhodeus amarus* and *Garra rufa* collected from Sıgırcı Lake, Gala Lake, Central Anatolia Region of Türkiye; Susuluk stream and Southern Türkiye with prevalence and mean intensity varying from 90% and 19.8%–8.3% and 1, respectively (Çolak 2013; Soylu 2013, 2014; Şimsek et al. 2018; Aydoğdu et al. 2020; Çağatay et al. 2022).

According to Yunchis (1998), the frequency of detection of a particular helminth parasite species in different host fishes or the same fishes is not constant but depends on conditions such as the geographical location of the study area, the season, host biology and the age and size of the host. Although the frequency of occurrence of a particular parasite species, which is usually detected in the same host or different fish host, is very similar in close habitats, it may differ in more distant habitats (Poulin 2007). On the basis of the above information, the fact that *A. escherichi* is an endemic species to Anatolia limits the recording of *C. complanatum* in other habitats (except Türkiye) within the same host fish species, thereby preventing the availability of comparative data on infection values. Furthermore, since *C. complanatum* is not known to occur in the same host fish across different habitats worldwide, no comparative conclusions can be drawn regarding the infection values of this parasite species. On the other hand, *C. complanatum* has been identified by various authors multiple times in several freshwater fish species in a wide geographical area worldwide (Çağatay et al. 2022). Although the infection prevalence values recorded for this species in the studies of all these authors were not similar to the value found in our study, surprisingly, Chung et al. (1995a) recorded an average intensity of infection of this parasite species similar to ours (7.5 ± 8.3 parasite/fish in this study) in *Microphysogobio yaluensis* (Mori, 1928) and *Carassius auratus* (L., 1758) (8.8 parasite/fish).

As to the infection results of this species in Türkiye, the prevalence of infection in the present study is higher compared to that reported by Soylu (2013) (Sığırcı Lake, Edirne), Çolak (2013) (Sığırcı Lake) and Aydoğdu et al. (2020) (Susurluk stream, Balıkesir) (15.4%, 13.1% and 13.75, respectively) and much lower than the prevalence reported by Soylu (2014) (Gale Lake, Edirne) and Çağatay et al. (2022) (53.85% and 90%, respectively). Our study revealed infection of *C*. *complanatum* metacercariae with an overall prevalence (combining both sexes) of 33.7%. This observation from the present investigation is not consistent with the finding of the aforementioned authors in Turkey. However, although we noted that the mean intensity level of infection was higher (7.5 ± 8.3 parasites/fish) compared to some authors’ data, others recorded a higher mean intensity level compared to our data. The findings above‐mentioned, as well as the reporting of *C. complanatum* in various fish specimens around the world (23 species) and in Türkiye (12 species), strongly indicate that *C. complanatum* is a widely distributed parasite species of freshwater fishes. This parasite does not make an evident host preference among fish species living in different environments. In addition, the differences in infection values reported by the above‐mentioned authors may be attributed to variations in food supply and the abundance of aquatic snails (the intermediate host) in the local habitats of the host fish. These differences may even be related to whether the locations where the study was conducted host aquatic fish‐eating birds that are essential for the completion of this parasite's life cycle. In addition, some of the fish species studied by these researchers may have been caught specifically from small and shallow tributaries of streams (this may contribute to the high prevalence levels). Such habitats, where water currents are slow or absent, provide ideal breeding grounds for local and migratory birds, including fish‐eating birds, and are also favourable for freshwater snails, which play the main role in completing the life cycle of this parasite.

In this study, the metacercariae parasites were collected across four different seasons, thus revealing a clear seasonal effect. The prevalence and mean intensity of infection varied from season to season throughout the entire research period (Table 1).

Seasonal variation in infection rates of *C. complanatum* in freshwater fishes has been studied worldwide. For example, Sharma et al. (2011) reported that the monthly prevalence and mean intensity values of infection of *C. complanatum* peaked in November/December (winter) in *Channa punctatus*, whereas these values were the lowest in July/August (summer). Alternatively, a study by Wang et al. (2017) investigated the seasonal effect on *C. complanatum* infection in 23 different fish species in Taiwan. The authors recorded infection of this species in only eight fish species and recorded no seasonal effect on the infection levels of this parasite these samples. Similarly, Juhasova et al. (2019) collected *P. fluviatilis* from five different localities in the Danube River, Slovakia (during 2 months only: October 2017 and April 2018). Although they reported the highest prevalence value of this parasite in two localities in October, the level decreased in April. The same authors could not record *C. complanatum* in October in the remaining three localities in their study. Likewise, Mahdy et al. (2022) reported the highest infection rate of this species in May in Nile tilapia. When the above‐mentioned seasonal infection findings of this species are compared with our study, it is found that the highest prevalence values recorded by Sharma et al. (2011) were compatible with our study, yet the highest mean intensity values recorded differ.

As for seasonal variations in infection rates in Türkiye, to date, this parasite species has been recorded in 12 different fish species, and its infection value was recorded in only 6 studies (Çolak 2013; Soylu 2013, 2014; Şimsek et al. 2018; Aydoğdu et al. 2020; Çağatay et al. 2022). Season‐related effects on the infection of *C. complanatum* were noted in the study of only one (Soylu 2013) of the above‐mentioned authors. Contrary to our finding, Soylu (2013) determined the maximum number of this species during summer in the perch (*P. fluviatilis*) in Sığırcı Lake.

The difference in the infection values of *C. complanatum* according to seasons is believed to be due to the difference in the time of exposure to the parasite, and the snail population growth. The number of trematode eggs shed into the water was due to rainfall and rising temperatures leading to cercarial production and accumulation of fish metacercaria (Sithithaworn and Haswell‐Elkins 2003). In addition, low water temperatures in winter increase the immune response and infection levels in fish. Abiotic factors (water temperature and precipitation) and biotic factors (age, sex, behaviour and mortality) may also contribute to the differences in infection values (Sherrard‐Smith et al. 2013). Furthermore, suitable hosts such as snails or birds may not be available in some of the localities of the above studies. In fact, if some of these localities have large water bodies, this may cause a decrease in metacercarial density in fish (Wang et al. 2017). As the authors, we think that the seasonal differences in the infection values of *C. complanatum* in our study are attributable to one or more of the factors mentioned above and we support the suggestions made by the aforementioned authors.

**FIGURE 7:**
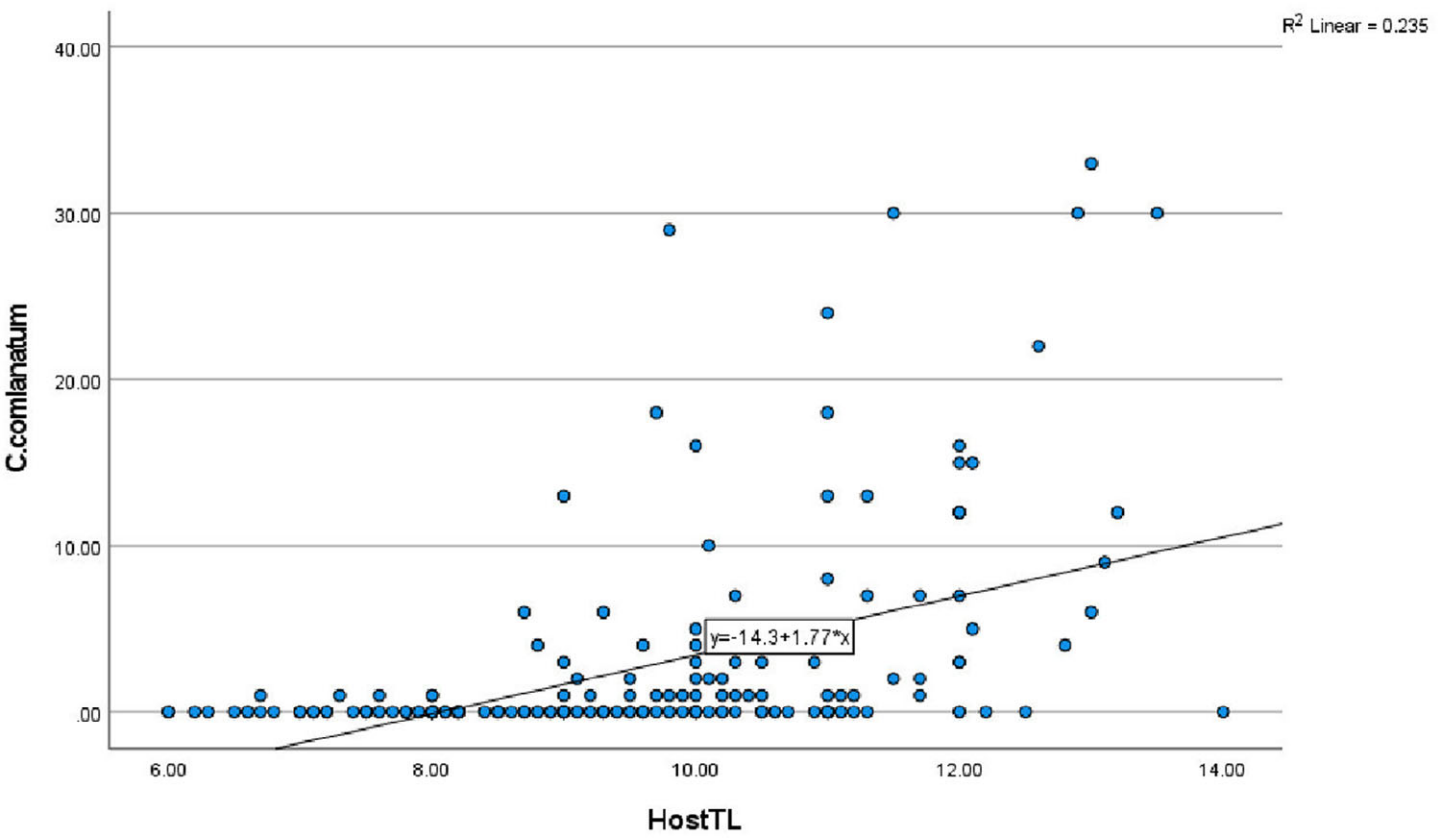
Regression plot of infracommunity: the number of *Clinostomum complanatum* metacercariae versus fish length.

We found a moderate (*r* = 0.534) but significant (*p* = 0.001) relationship between fish host length and metacercarial infection levels in *A. escherichii* (Figure 7). Similarly, Mahdy et al. (2022) also reported a positive correlation between the number of collected metacercariae and the length of *Zaccopachy cephalus*, whereas the same authors did not find a significant relationship between the length of the fish host *Acrossocheilus paradoxus*, *Zacco barbata* and *Z. platyus* and the infection by the parasite species. Moreover, the relationship between the prevalence of *C. complanatum* metacercariae and the length of host fish is studied by Maleki et al. (2018). They reported a positive relationship between the length of *Capoeta damascina* and the prevalence of *C. complanatum*. Contrary to these findings, Silva‐Souza and Ludwing (2005) found no host length–related effects on the intensity of this parasite infection. These authors suggested that this might be due to the fact that larger fish have greater surface area, allowing cercariae to attach more readily. Additionally, the larger fish tend to be older and therefore harbour more parasites. The findings of our study in this context also support these suggestions.

Because female and male host fish individuals have different biological characteristics, infection levels of parasite species vary from one sex to the other. Exactly in parallel with this information, in our study, the overall prevalence, mean intensity and abundance of *C. complanatum*, observed were higher in females compared to males (Table 1). The relationship between the sex of host fish and *C. complanatum* infection has been studied by Maleki et al. (2018). Contrary to our finding, they found no host sex‐related effects on the prevalence of *C. complanatum* on four fish species (*Alburnus mossulensis*; *C. damascina*, *G. rufa* and *S. cephalus*). Additionally, we have been unable to find any studies in the literature on the influences of host sex on *C. complanatum* infections in freshwater fishes. On the other hand, we generally know that some authors (Aydogdu et al. 2018; Mnisi 2017) have found higher digenean infection in male fish, whereas others (Aydoğdu et al. 2015; Emre and Kubilay 2019) reported higher infection in female fish. They concluded that this was due to different biological characteristics between the sexes of the host which could lead to one sex being more parasitized than the other. This may indicate that female and male individuals of *A. escherichii* exhibit different behaviours, habitat uses, environmental conditions or exposure times to the parasites.

Regarding the possible zoonotic risk of this parasite species, *C. complanatum* metacercariae may pose a risk to humans through consumption of infected fish. Therefore, it was concluded that consumption of *A. escherichii* and the 12 fish species in which this parasite was reported in Türkiye, many of which have economic value, may cause zoonotic infection to consumers. To date, however, to our knowledge, no cases of pharyngitis and laryngitis caused by this parasite species have been reported in humans in Türkiye. Human infections have been recorded mostly in Asian countries with a tradition of eating raw fish (Chai and Jung 2017). Worldwide awareness of the zoonotic potential of *C. complanatum* in fish is increasing day by day (Menconi et al. 2020). Additionally, the number of hosts infected by this parasite species is one of the biggest threats to its potential global spread. The number of studies reporting the presence of this parasite in Türkiye is steady. The fish species in which this parasite has been recorded in Türkiye have been used in traditional local cuisine, but generally not as raw fish. However, the zoonotic risk caused by this parasite species may also emerge in Türkiye if the tradition of consuming raw fish increases. Finally, these and similar studies on the effects of clinostomiasis on fish hosts provide important data for controlling fish‐borne zoonotic diseases caused by this parasite species that threatens human health.

Amplification of 1021 bp of the ITS gene area was performed in our study, and the results were compared to the GenBank data. Here, the ITS gene marker was used for molecular identification and DNA sequencing, allowing for the identification of the metacercariae as *C. complanatum* (Figure 4). Our sequences clustered within the BI phylogenetic tree (PP = 1.00) into the *C. complanatum* species. Previous studies have demonstrated the utility of the ITS region, the 18S rRNA and the mtCOI in supporting morphological data for detecting new species within the genus *Clinostomum*, in the molecular identification of species and in studying genetic variation and relationships between species (Acosta et al. 2016; Chung et al. 1995b; Locke et al. 2015; Şimsek et al. 2018).

## 5 Conclusion

To the best of our knowledge, this is the first record of *C. complanatum* metacercariae in the endemic Anatolian fish species, *A. escherichii* in Türkiye, as well as from this previously unexplored host and region. Thus, it represents a new locality for the distribution of *C. complanatum*. As a result, we believe that this parasite can be transported to other countries through international fish trade and migratory birds and may be reported from different countries in the future. We consider it necessary to reveal the geographical distribution of the parasite in order to minimize its spread and, if possible, eliminate it. This study contributes to this field in this context. Furthermore, this study provides valuable information for the molecular characterization of this species collected from Türkiye. In addition, the sequence data of *C. complanatum* from *A. escherichii* were reported to GenBank for the first time from Sarısu stream. Moreover, this study provides further insight into how the infection parameters of this species vary with seasons and host fish length and sex. Additionally, histopathological effects of this parasite species on host fish tissues were identified. In this regard, this study is the first to determine the histopathological effects of *C. complanatum* on the tissues of freshwater fish species in Türkiye. Finally, considering the increase in zoonotic diseases worldwide, studies on helminth parasite species with zoonotic potential are of great importance. Therefore, investigating the transmission of helminth parasite species with zoonotic potential to other fish species along with ongoing and new research findings is crucial for understanding the causes of zoonotic diseases. This study also contributes to the knowledge in this field.

## Author Contributions

**Nurten Aydoğdu**: writing – review and editing, writing – original draft, visualization, investigation, formal analysis, data curation, conceptualization. **Simonetta Mattiucci**: writing – review and editing **Marialetizia Palomba**: writing – review and editing **Ali Aydoğdu**: writing – review and editing, investigation, data curation, conceptualization. **Çiğdem Ürkü**: writing – review and editing, investigation, formal analysis. **H. Emre Yılmaz**: writing – review and editing, formal analysis, investigation. **I. Tülay Çağatay**: writing – review and editing, formal analysis, investigation. **Sadi Aksu**: investigation.

## Ethics Statement

No ethical approval was required, as this study did not involve clinical trials or experimental procedures. During the study, no treatment/experiment was implemented on live animals. All sampling and laboratory work on fish have complied with the Republic of Türkiye Ministry of Agriculture and Forestry animal welfare laws.

## Conflicts of Interest

The authors declare no conflicts of interest.

### Peer Review

The peer review history for this article is available at https://www.webofscience.com/api/gateway/wos/peer‐review/10.1002/vms3.70270.

## Data Availability

The authors declare that they do not have any shared data available.
